# A Case of Schizencephaly and Septo-Optic Dysplasia Presenting with Anterior Encephalocele

**Published:** 2012

**Authors:** Kaveh FADAKAR, Sahar DADKHAHFAR, Arash ESMAEILI, Zarrintaj KEYHANIDOUST

**Affiliations:** 1 Medical Student, Department of Medicine, School of Medicine, Tehran University of Medical Sciences, Tehran, Iran; 2 Associate Professor of Pediatric Neurology, Department of Pediatrics, School of Medicine, Tehran University of Medical Sciences, Tehran, Iran

**Keywords:** Encephaloceles, Septo-optic dysplasia, Childhood, Nervous System Diseases, Schizencephaly

## Abstract

Schizencephaly is a rare central nervous system disorder with variable presentations. Here we report a patient with a huge bilateral schizencephaly and septo-optic dysplasia presenting with anterior encephalocele.

## Case Report

A 6-month-old infant was referred to our center with the chief complaint of a 2cm 3cm mass located at the root of the nose. The mass was first identified at birth with an initial misdiagnosis of lacrimal duct stenosis and the cyst had increased in size by that time. No history of lacrimation, rhinorrhea, hypertelorism, strabismus or other ophthalmologic manifestations were obtained. The parents reported that the mass enlarged during crying.

The infant was the fourth child of four children of relative parents and was born by an uneventful natural vaginal delivery with the birth weight of 2750 grams. Maternal history for prenatal disease was negative except for a large cerebral cyst reported by ultrasound in the third trimester.

At the time of referral to our center, the patient’s weight was 6 kg with no signs of failure to thrive. The head circumference was 40cm with a 2cm×2cm open fontanelle. He had head control since 4 months of age and could sit with help and also reach for objects, hold bottle on his own and was able to laugh and bubble at the time of examination. Otherwise, neurological and systematic assessment was otherwise normal.

**Fig 1:**
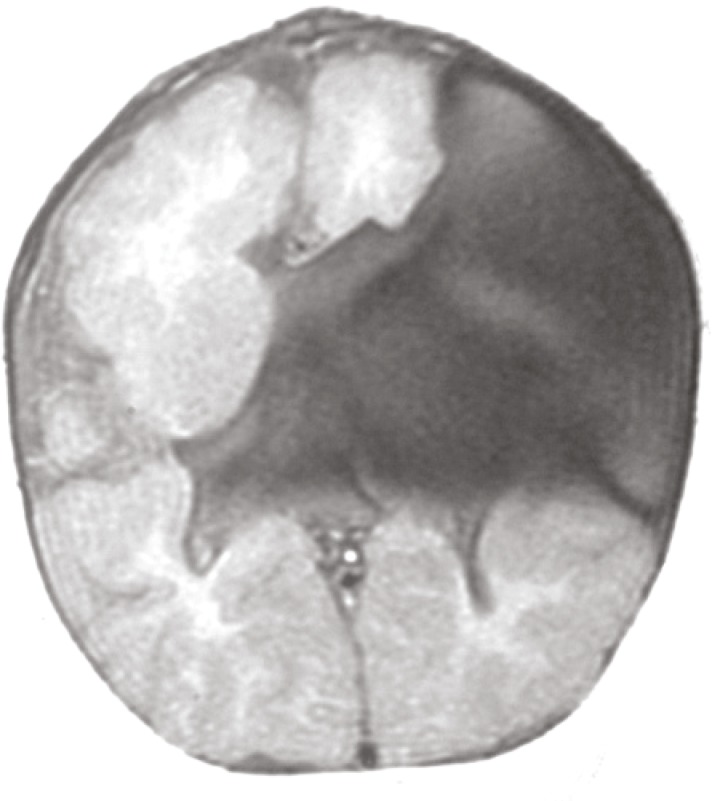
T1 weight haly on the left side associated with cystic extra axial structures and fine closed-lip schizencephaly on the right side

**Fig 2:**
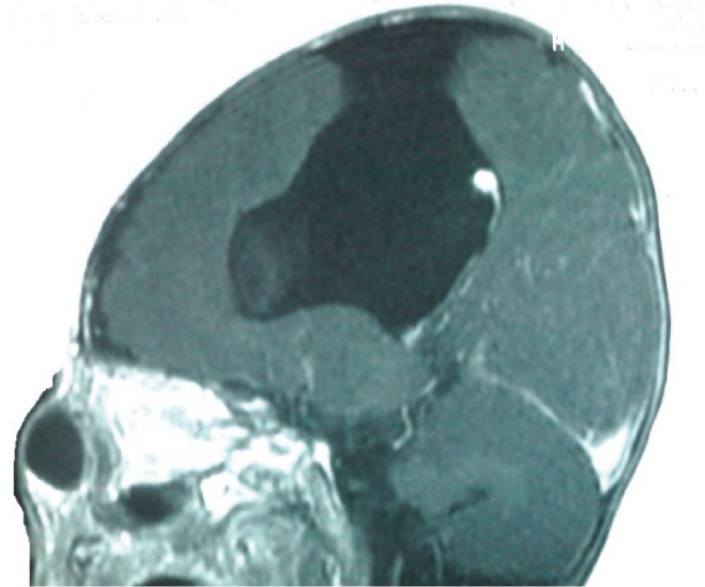
Axial T1 weighted MRI shows anterior skull-based meningocele

Since the mass grew larger while crying, there was suspicion of its possible connection with the intra cranial space. Based on the above mentioned fact and that the patient had microcephaly plus slight developmental impairment we performed a brain magnetic resonance imaging which revealed large open lip schizencephaly on the left side associated with cystic extra axial structures, fine closed lip schizencephaly on the right side, septo-optic dysplasia, dysgenesis or partial agenesis of the corpus callosum and opening of the foramen cecum toward left nasal root cystic structures suggesting anterior skull based encephalocele (Figures 1,2).

**Fig 3:**
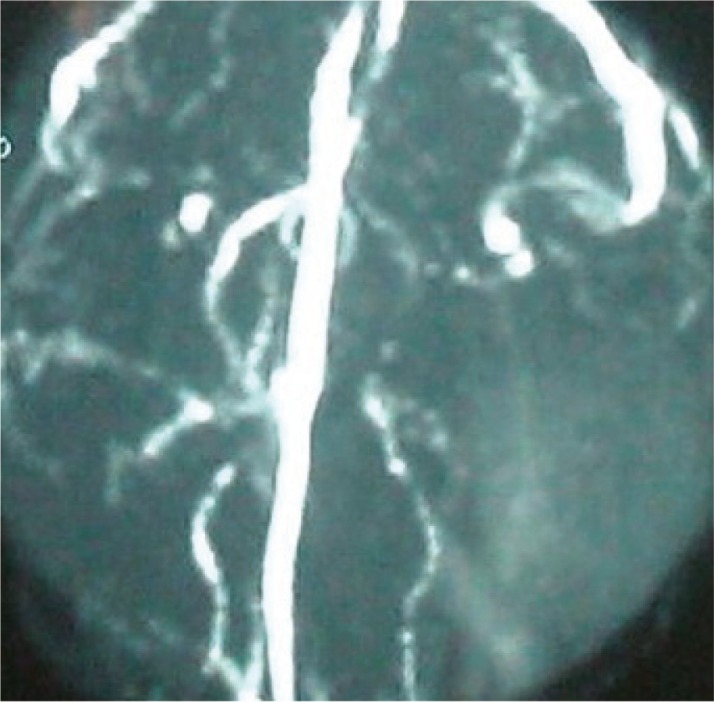
MR angiography reveals hypovascularity and middle cerebral artery displacement

In addition, MR angiography results showed main vessel displacement, especially the middle cerebral artery branch on the left side and some hypovascularity was noted related to large cystic structures and described abnormalities (Figure 3).

The patient had myoclonic jerks during sleep which was matched with the abnormal electroencephalogram pattern. The myoclonus was controlled after administration of primidone. Ophthalmologic examination was normal. Auditory brain response revealed moderate to severe hearing defect in the left ear.

In order to investigate the possible existence of other midline lesions, we performed an echocardiography and abdominal sonography and both of them were normal. Laboratory exploration did not add other findings worthy of note.

The child was admitted to the neurosurgical ward for correction of the encephalocele. Informed consent was obtained from the patient’s parents under the guidelines of the ethics committee of Tehran University of Medical Sciences.

## Discussion

Schizencephaly was first described by Yakovlev and Wadworth in 1946 (1, 2). There are two types of schizencephaly. One of them is closed-lip clefts in which the clefts are in contact with each other and are covered by gray matter. The other type is the open-lip cleft in which the lesion occupies the space between the ventricles and the subarachnoid space. There are also subdivisions to such typing like unilateral or bilateral lesions. The open-lip cleft can also be divided into small, medium and large size.

The exact etiology of such a defect is still unclear, but it seems that a prenatal insult that causes inability of neurons to migrate during late cortical ontogenesis might be a reason. Many factors impair the migration of neurons like mutation of EMX2 gene (3) or vascular prenatal injuries (4).

Clinical manifestations of schizencephaly include seizure, motor deficit, abnormal cognitive development and impairment of language skills. Although a wide range of variable clinical manifestations are seen in these patients, open, large and bilateral clefts are usually related to severe disease (5). According to what was mentioned before we expected more significant motor deficit and developmental retardation in our patient; however, prenatal cortical re-organization can explain lack of retardation in this case. This hypothesis is supported by functional MRI studies which demonstrated extra activity of intact hemisphere to cover the damaged hemisphere’s function (6). Schizencephaly is accompanied by neurological complications like agenesis of the corpus callosum and/or septum pellucidum and also ventriculomegaly and arachnoid cysts.

MRI findings revealed agenesis of septum pellucidum and relative hypoplasia of the optic nerve in this case. These are two of three characteristic signs of septo optic dysplasia (triad of absence of the septum pellucidum and optic nerve abnormalities in association with pituitary dysfunction). Septo optic dysplasia (SOD) was first explained by Reeves in 1941 according to the parallel lack of septum pellucidum in patients with optic nerve disorders (7). Subsequently, existence of concurrent hypophysial abnormality was illustrated in these patients (8). Although the diagnosis is confirmed by observing at least two signs of this triad, we can suggest the diagnosis of SOD for our patient despite the normal ophthalmological examinations. In fact, this case might be one of the milder presentations of such disorders which can be categorized into a wide range (9). Coexistence of schizencephaly and SOD is an uncommon yet known finding that may be related to the primary defect leading to schizencephaly. Schizencephaly may also cause dysgenesis of the previously developed septum pellucidum or the optic nerve (10). Nevertheless, there is no report of simultaneous schizencephaly, SOD and anterior encephalocele particularly with the lack of profound developmental disorders seen in this child. Although there was some bilateral senorineuronal auditory dysfunction in our patient, lack of motor function retardation and other midline defects are worthy of attention. As stated by many papers, these patients are exposed to experience cognitive and language disorders along with episodes of seizures which may not be evident at presentation, but have a potency of showing themselves in the future. Therefore, close follow up of this child is necessary for early detection of these disorders.
